# Adjunctive High Frequency Left Dorsolateral Prefrontal Cortex (L-DLPFC) rTMS Reduces Craving, Anxiety, and Depression in Inpatient Addiction Rehabilitation: A Pilot Service Evaluation

**DOI:** 10.1192/j.eurpsy.2026.11752

**Published:** 2026-07-31

**Authors:** R. Iosub, N. Airey, N. Maynard

**Affiliations:** 1 AIM Neuromodulation; 2 Broadway Lodge Rehabilitation Clinic, Weston-super-Mare, United Kingdom

## Abstract

**Introduction:**

Addiction is often accompanied by high rates of comorbid depression and anxiety, complicating treatment outcomes. Repetitive transcranial magnetic stimulation (rTMS) is an established therapy for depression and has shown promise in reducing drug craving when applied to the L-DLPFC.

**Objectives:**

We examined the effect of adjunctive rTMS to the L-DLPFC on the severity of cravings, anxiety and depression in patients undergoing inpatient addiction rehabilitation.

**Methods:**

We conducted an observational pilot study in a residential addiction treatment facility. **Participants:** 12 inpatients with substance use disorders (alcohol, opioids, stimulants, cannabis) or behavioral addiction (sex addiction), received standard rehabilitation and add-on rTMS targeting the L-DLPFC.

**Intervention:** High frequency rTMS was delivered as per CE approved Magventure Addiction protocol adapted for the inpatient setting. 15 Hz stimulation to the L-DLPFC at 100% of resting motor threshold which provides a total of 2400 pulses per session (intended to modulate craving and mood-related circuits). Each patient received between 20 and 30 sessions over 4-6 weeks.

**Outcome Measures:** Craving severity (self-rated 0–10 VAS) and Hospital Anxiety and Depression Scale were administered every 10 treatment sessions, tracked from baseline to the end of rTMS treatment. We compared baseline and final scores and performed paired statistical tests to assess improvements.

**Results:**

Out of 12 patients, 10 completed the full rTMS course (2 withdrew before final assessment). Marked improvements in cravings and affective symptoms were observed in completers:

**Craving:** Mean severity dropped from 4.7 at baseline to 1.2 at final assessment (≈74% reduction, *p*<0.01). By treatment end, 8 of 10 patients reported craving virtually extinguished.

**Anxiety:** Mean anxiety ratings fell from 12.0 at baseline to 6.8 post-rTMS (*p*<0.01). This reflects a shift from moderate to mild levels on the anxiety scale, indicating a clinically significant relief of anxiety symptoms.

**Depression:** Mean depression scores declined from 8.5 to 2.9 (*p*<0.01), an improvement of about 66%. All patients’ post-treatment depression scores fell into the minimal range, effectively resolving major depressive symptoms in this cohort.

No serious adverse effects were noted.

**Conclusions:**

High frequency rTMS targeting the L-DLPFC was associated with significant reductions in craving and improvements in anxiety and depression in patients undergoing residential addiction treatment. These findings align with emerging evidence and suggest that rTMS may be a valuable augmentation to standard addiction rehabilitation, addressing both the addictive drive and co-occurring emotional symptoms. Larger controlled studies are warranted to confirm these benefits and to evaluate the impact on long-term recovery.

**Disclosure of Interest:**

None Declared

